# Mediators of Gut Mucosal Immunity and Inflammation

**DOI:** 10.1155/2015/765303

**Published:** 2015-04-19

**Authors:** Ishak Ozel Tekin, H. Barbaros Oral, Ronit Shiri-Sverdlov

**Affiliations:** ^1^Department of Immunology, School of Medicine, Bulent Ecevit University, 67600 Zonguldak, Turkey; ^2^Department of Immunology, School of Medicine, Uludag University, Gorukle, 16059 Bursa, Turkey; ^3^Department of Molecular Genetics, Maastricht University (MUMC), Universiteitssingel 50, 6229 ER Maastricht, Netherlands

The gastrointestinal tract is continuously exposed to foreign antigens such as those derived from food and microbiota of gut. Gut flora is an important entity of human body that plays crucial role in healthy immune system and various immunological disorders. Exploration of interactions between gut flora and immune system is an important area of current investigations. Cytokines and chemokines are the integral component of the adaptive and innate immune response in the gastrointestinal system. Cytokines are involved in a variety of immunological, inflammatory, and infectious diseases. Chemokines are a family of small cytokines or proteins secreted by numerous cells. The major role of chemokines is to guide the migration of particular cells. The mediators of gut mucosal immunity and inflammation are not limited to chemokines and cytokines. There are many inflammatory and anti-inflammatory molecules derived from gut mucosal endothelial cells and leucocytes.

In this special issue, we report findings regarding the mediators of gut mucosal immunity and inflammation. The papers have been contributed by a number of experts in the field and include both review articles that provide an overview of the work conducted to date and original articles reporting recent developments. This special issue contains 19 papers, representing original research articles, a clinical research article, and reviews. In order to highlight the translational relevance, several papers are focused on colitis mechanisms as well as clinical evidence related to inflammatory bowel diseases. Some papers offer important relationship between microbiota, probiotics, functional foods, and gut mucosal immunity and inflammation.

We hope that these papers will be beneficial for clinicians and researchers in understanding of gut mucosal immunity and inflammation on health or diseases. Each of the papers in this series is briefly highlighted as follows.

Y.-C. Hou et al. in “Glutamine Supplementation Attenuates Expressions of Adhesion Molecules and Chemokine Receptors on T Cells in a Murine Model of Acute Colitis” describe an animal study that investigates the effect of glutamine on the expression of some adhesion molecules and chemokine receptors on T cells. According to this paper, glutamine may ameliorate the inflammation of colitis via suppression of T-cell migration.

H. Rajkumar et al.'s “Effect of Probiotic (VSL#3) and Omega-3 on Lipid Profile, Insulin Sensitivity, Inflammatory Markers and Gut Colonization in Overweight Adults: A Randomized, Controlled Trial” is a clinical study that examined the effect of VSL#3 and omega-3 on some biochemical and inflammatory markers and gut colonization in overweight human adults. In this study VSL#3 alone improves atherogenic biochemical profile and insulin sensitivity and changes gut microbiota. Omega-3 has a similar effect with probiotics but it has no effect on gut microbiota. The combination of VSL#3 and omega-3 has more pronounced effect on HDL, insulin sensitivity, and hsCRP.

“Intestinal Mucosal Barrier Is Injured by BMP2/4 via Activation of NF-*κ*B Signals after Ischemic Reperfusion” by K. Chen et al. is a research article reporting the effect of bone morphogenic proteins (BMP 2 and BMP 4) on intestinal mucosal barrier for an ischemia-reperfusion model. In this study, BMP2 and BMP4 can directly activate NF-*κ*B, induce the expression of some inflammatory cytokines in the intestinal epithelial cells, and decrease the expression of the tight junction protein occludin, which could result in disruption of the intestinal barrier.

“An Overview of the Role of Innate Lymphoid Cells in Gut Infections and Inflammation” by S. Sedda et al. is a review about innate lymphoid cells in the gut. In this review, the authors summarized the current knowledge on the distribution of ILCs in the intestinal mucosa, with particular focus on their role in the control of both infections and effector cytokine response in immune-mediated pathologies.

The important role of IL-35 and IL-37 in IBD patients is presented by Y. Li et al. in the research article “The Possible Role of the Novel Cytokines IL-35 and IL-37 in Inflammatory Bowel Disease.” The study focuses on IL-35, IL-37, and IBD. According to the authors, serum IL-35 and IL-37 might be potentially novel biomarkers for IBD. And the upregulation of encoding genes to intestinal IL-35 and IL-37 proteins may provide a new possible target for the treatment of IBD.

A. K. Kumawat et al. presented a paper entitled “An In Vitro Model to Evaluate the Impact of the Soluble Factors from the Colonic Mucosa of Collagenous Colitis Patients on T Cells: Enhanced Production of IL-17A and IL-10 from Peripheral CD4+ T Cells.” This model reveals implications of soluble factors from collagenous colitis mucosa on peripheral T cells, enhancing their production of both pro- and anti-inflammatory cytokines.

K. S. Brown et al.'s “Tumor Necrosis Factor Induces Developmental Stage-Dependent Structural Changes in the Immature Small Intestine” is a research article reporting the effects of TNF on structural changes in the immature small intestine. In this study, the researchers examine acute, brief, or chronic exposures of TNF in neonatal and juvenile mice. In this model, TNF-induced blunting caused by feeding-induced or other chronic inflammation could subsequently decrease the distance between the luminal contents of the intestine and the lamina propria. This shortening of distance would greatly increase the ability of bacteria to reach the crypt and infiltrate the intestine. The authors observed also the other effects of TNF such as depletion of the mucous layer and degranulation of Paneth cells, which may allow for easier bacterial penetration into the intestinal lamina propria, leading to the inflammatory response and coagulation necrosis characteristic of NEC.

“The Impact of ATRA on Shaping Human Myeloid Cell Responses to Epithelial Cell Derived Stimuli and on T-Lymphocyte Polarization” by A. Chatterjee et al. is a research article about the importance of ATRA on triangle of myeloid cell, epithelial cell, and T cell.

Various immune cell infiltrations in the epithelium and lamina propria are seen in microscopic colitis immunopathology. “Enhanced Levels of Chemokines and Their Receptors in the Colon of Microscopic Colitis Patients Indicate Mixed Immune Cell Recruitment” by S. Gunaltay et al. is a research article. The study focuses on the chemokines and their receptor levels in the colon of microscopic colitis patients. The results of this study expand the current understanding of the involvement of various immune cells in MC immunopathology and endorse chemokines as potential diagnostic markers as well as therapeutic candidates. Moreover, this study further supports the hypothesis that CC and LC are two different entities due to differences in their immunoregulatory responses.

The review entitled “Transcriptional Regulators of Claudins in Epithelial Tight Junctions” by N. Khan and A. R. Asif focuses on the transcriptional regulators of claudins. This review indicates that altered expression of claudins family proteins in tight junctions plays a key role in numerous abnormalities like cancers, IBDs, and leaky diarrhea and a better understanding of their regulatory mechanism could help in designing innovative therapeutic strategies.

J. Michalkiewicz et al. in “Innate Immunity Components and Cytokines in Gastric Mucosa in Children with* Helicobacter pylori* Infection” evaluate innate immunity in gastric mucosa in children with HP infection. This study showed that* H. pylori* infection in children resulted in mRNA upregulation of IL-6, IL-10, TNF-*α*, IFN-*γ*, and CD163 and unchanged expression of MyD88, TLR2, and TLR4 mRNA in the gastric mucosa. According to the authors, these findings are associated with* H. pylori* driven immune manipulation.

“Moderate Exercise Training Attenuates the Severity of Experimental Rodent Colitis: The Importance of Crosstalk between Adipose Tissue and Skeletal Muscles” is a research article about rodent experimental colitis realized by J. Bilski et al. In this study, diet induced obesity delays the healing of experimental colitis. Release of myokines in trained animals contributes to improvement of intestinal healing. The authors recommend an exercise program for IBD patients.

S. O'Sullivan et al. in “Matrix Metalloproteinases in Inflammatory Bowel Disease: An Update” reviewed the effects of matrix metalloproteinases in IBD. This review describes new roles of MMPs in the pathophysiology of IBD and suggests future directions for the development of treatment strategies in this condition.

“*Enterococcus faecium* NCIMB 10415 Modulates Epithelial Integrity, Heat Shock Protein, and Proinflammatory Cytokine Response in Intestinal Cells” by S. Klingspor is a research article about relationship between* Enterococcus faecium* NCIMB 10415 and intestinal cells. The effects of* E. faecium* observed in this study indicate a protective effect of this probiotic in acute intestinal inflammation induced by ETEC.

M. Endale et al. in “Central Role of Gimap5 in Maintaining Peripheral Tolerance and T Cell Homeostasis in the Gut” reviewed the role of GTPase of immunity-associated protein 5 (Gimap5) in maintaining peripheral T-cell tolerance in the gut. The authors discuss how defects in Gimap5 function impair immunological tolerance and lymphocyte survival and ultimately drive the development of CD4+ T cell-mediated early-onset colitis in this paper.

“Oxidative Stress in Patients with Alzheimer's Disease: Effect of Extracts of Fermented Papaya Powder” is a review article written by M. Barbagallo et al. This review focuses on the effects of fermented papaya in the patients with Alzheimer diseases.

Y. Kurashima et al. in “Pathophysiological Role of Extracellular Purinergic Mediators in the Control of Intestinal Inflammation” reviewed the recent findings regarding the pathophysiological role of purinergic mediators in the development of intestinal inflammation.

“Gut Inflammation and Immunity: What Is the Role of the Human Gut Virome?” by A. Focà is a review article about the role of human gut virome on gut inflammation and immunity. The authors reviewed recent evidence on the viruses found in the gastrointestinal tract, discussing their interactions with the resident bacterial microbiota and the host immune system, in order to explore the potential impact of the virome on human health.

Finally, “Claudin-4 Undergoes Age-Dependent Change in Cellular Localization on Pig Jejunal Villous Epithelial Cells, Independent of Bacterial Colonization” by J. A. Pasternak et al. showed that* FcRn* gene (FCGRT) was minimally expressed in 6-week-old gut and newborn 24 jejuna but it was expressed at significantly higher levels in the ileum of newborn piglets. pIgR was highly 25 expressed in the jejunum and ileum of 6-week-old animals but only minimally in neonatal gut. According to the authors, CLDN4 transcript abundance and CLDN5 transcript abundance were conserved in jejunum and ileum in age-matched animals and that striking differences in CLDN4 expression did not occur in either region of the gut with age. CLDN5 showed significantly higher expression in the jejunum and ileum from the 24-hour-old animals relative to the older animals.

We sincerely hope that the present special issue may provide useful information to understand the mediators of gut mucosal immunity and inflammation. We hope that the reader will find some novel input for future researches.

